# In Vitro Testing of the Virus-Like Drug Conjugate Belzupacap Sarotalocan (AU-011) on Uveal Melanoma Suggests BAP1-Related Immunostimulatory Capacity

**DOI:** 10.1167/iovs.64.7.10

**Published:** 2023-06-05

**Authors:** Sen Ma, Ruben V. Huis In't Veld, Alexander Houy, Marc-Henri Stern, Cadmus Rich, Ferry A. Ossendorp, Martine J. Jager

**Affiliations:** 1 Department of Ophthalmology, Leiden University Medical Center (LUMC), Leiden, The Netherlands; 2 Department of Radiology, Leiden University Medical Center (LUMC), Leiden, The Netherlands; 3 Department of Immunology, Leiden University Medical Center (LUMC), Leiden, The Netherlands; 4 Inserm U380, DNA Repair and Uveal Melanonoma (D.R.U.M.), Equipe labelliseé par la Ligue Nationale Contre le Cancer, Institut Curie, PSL Research University, Paris, France; 5 Aura Biosciences, Inc., Cambridge, Massachusetts, United States

**Keywords:** eye disease, oncology, photodynamic therapy, uveal melanoma (UM), immunotherapy, AU-011, belzupacap sarotalocan

## Abstract

**Purpose:**

The virus-like drug conjugate belzupacap sarotalocan (AU-011), currently under clinical investigation for first-line treatment of primary uveal melanoma (UM), shows enhanced tumor specificity by targeting heparan sulfate proteoglycans (HSPG). Such a treatment may potentially lead to systemic immune responses. We studied the potential of AU-011 treatment to induce immunogenic cell death as the first step to induce systemic immunity.

**Methods:**

We determined binding and uptake of AU-011 in ten primary and metastatic UM cell lines. The subcellular location of AU-011 was assessed by fluorescence microscopy. Following light activation (wavelength 690 nm) of AU-011, the half-maximal effective concentration (EC_50_) of AU-011 treatment and exposure of damage-associated molecular patterns (DAMPs) were assessed using flow cytometry. DAMPs were measured by RNAseq.

**Results:**

Fluorescence microscopy revealed most of the AU-011 was present in the cytoplasm. AU-011 binding and uptake by UM cells increased over time, with a lower uptake in BAP1-negative than in BAP1-positive cell lines. AU-011 activation induced cell death across all UM cell lines with EC_50_ values at picomolar concentrations. The AU-011 concentration and total light dose (J/cm^2^) were the most important parameters for the observed cytotoxicity. Finally, light-activated AU-011 induced exposure of DAMPs calreticulin (CRT) and HSP90. CRT exposure by light-activated AU-011 as well as CRT RNA exposure were lower in BAP1-negative compared to BAP1-positive UM cell lines.

**Conclusions:**

AU-011 treatment at low picomolar range induces immunogenic cell death in all 10 UM cell lines. The in vitro cytotoxicity was accompanied by exposure of DAMPs (HSP90 and CRT), suggesting AU-011 may contribute to the development of systemic immunity and be a suitable candidate for combination with immunotherapy in vivo. AU-011 treatment was more effective against BAP1-positive cell lines, with a lower EC_50_ and higher CRT exposure.

Uveal melanoma (UM) is the most common primary ocular malignancy in adults and is often lethal.^1^^–^^4^ For large tumors, removal of the eye (enucleation) may be necessary as saving vision may then not be feasible. Often, local irradiation with a radio-active plaque or proton beam therapy is an effective treatment but may lead to serious impairment of vision.^5^^–^^7^ Because of the side effects, these treatments are usually not used for suspicious or indeterminate pigmented choroidal lesions, which up to now are often left untreated. Currently, treatment with AU-011 (a virus-like drug conjugate) is being investigated in clinical trials in these indeterminate pigmented choroidal lesions and small UM.^8^^,^^9^ Even after successful treatment, UM metastasizes in up to 50% of patients with UM, especially to the liver. When metastases are clinically evident, prognosis for patients with UM drastically decreases to a median survival of approximately 4 to 15 months.^10^^,^^11^ The discovery of immune checkpoint inhibitors (ICIs) has given many patients with cancer an improved chance of survival by relieving the tumor's inhibition of the immune system. Unfortunately, ICIs (including anti-PD-1 and anti-CTLA-4 antibodies) have led to some but not a dramatic improvement in survival of patients with UM metastases.^12^^–^^14^ The main reasons for the lack of efficacy may be related to the low mutational burden of UM and the immune privilege of the eye, which may inhibit local immune responses.^15^^,^^16^

The development of metastases is strongly related to genetic changes, especially to loss of one chromosome 3 and loss of expression of BRCA1-associated protein-1 (BAP1)^17^^–^^19^: up to 50% of primary UM has a biallelic inactivation in the BAP1 gene, which is associated with a highly increased risk of metastases.^20^ The BAP1 gene is located on chromosome 3 and mutations in this gene are often accompanied by loss of the other chromosome 3, leading to lack of expression. BAP1 is a nuclear deubiquitinating hydrolase that plays a crucial role in DNA damage repair, regulation of apoptosis, and protein deubiquitination.^21^^,^^22^ Loss of BAP1 expression is associated with an increased density of tumor-infiltrating lymphocytes and tumor-associated macrophages, which may be involved in immune suppression.^18^^,^^20^^,^^23^

For these reasons, we hypothesized that in order to overcome the inhibition of the antitumor immune response, enhancement of the initial immune response may be needed to obtain a significant antitumor effect in UM. Such a response may start in the eye and may be achieved by first damaging tumor cells with photodynamic therapy (PDT). PDT is a clinically applied, minimally invasive tumor ablation method in which the energy of light is used to produce free radicals, such as reactive oxygen species (ROS). The presence of ROS may affect tumor cells directly or indirectly through vascular damage. In addition, cell death after PDT can initiate immunogenic cell death (ICD), a mode of cell death that is often accompanied by the exposure and release of damage-associated molecular patterns (DAMPs). DAMPs induced by PDT include calreticulin (CRT), high mobility group box 1 (HMGB1), and heat shock proteins (HSPs). HSP70 and HSP90 have been shown to induce maturation of antigen-presenting cells (APCs) and initiate an immune response.^24^^,^^25^ A recent study showed that in a dual-tumor animal model, local PDT led to eradication of treated tumors and an enhancement of tumor-specific (CD8^+^) T-cell responses that inhibit the growth of distant untreated tumors.^26^ A study on B16F10 cells showed that treatment of an intraocular tumor with a combination of PDT and a rho kinase indeed partly protected mice from developing lung metastases.^27^ A possible mechanism behind this observation is that PDT induces a mode of cell death that allows access to previously inaccessible tumor (neo)antigens. Combining PDT with ICIs can further enhance these effects, eradicating local as well as distant tumors.^27^^–^^29^

Photosensitizers are molecules that can actively convert the energy of light to ROS. AU-011 contains a photosensitizer and is being investigated for the treatment of indeterminate lesions and small UM (ClinicalTrials.gov: NCT02422979; Ref. 8). AU-011 is composed of two parts: a virus-like particle that specifically targets heparan-sulfate proteoglycans (HSPGs), which are modified and overexpressed specifically on malignant cells, and a phthalocyanine photosensitizer, which can be excited by light with a wavelength of 690 nm.^9^^,^^30^ We recently showed that treatment with AU-011 and ICIs induced an abscopal effect in a murine model using MC38 cells.^28^ Prior in vitro tests showed that AU-011 worked well on UM cell lines.

In the present study, we determined whether AU-011 initiated immunogenic cell death in UM cell lines and compared the effect of AU-011 treatment in relation to BAP1, comparing BAP1-negative and BAP1-positive UM cell lines. Our data show that BAP1-negative cells are less prone to undergo immunogenic cell death than BAP1-positive cells and display less DAMPs. Our data may inspire future research to enhance the treatment response for patients with UM.

## Materials and Methods

### Cell Lines and Cell Culture

Tumor cell lines were cultured under 5% CO_2_ at 37°C in RPMI1640 and IMDM medium (Life Technologies, Europe bv, Bleiswijk, The Netherlands), supplemented with 10% or 20% heat-inactivated fetal calf serum (Greiner Bio-one, Alphen aan den Rijn, The Netherlands) and 1% penicillin/streptomycin (Life Technologies).

Cell line 92.1 came from our laboratory (LUMC, Leiden, The Netherlands), OMM1 was a gift from Dr. Gre Luyten, Rotterdam, and cell lines Mel270, Mel285, OMM2.3, and OMM2.5 were a gift from Dr. Bruce Ksander (Schepens Eye Research Institute, Boston, MA, USA). Cell lines MP38, MP46, MM28, and MM66 were a gift from Dr. Sergio Roman-Roman and Dr. Didier Decaudin, Curie Institute, Paris, France.^31^^–^^35^

Cell line characteristics:

• Primary UM cell lines: 92.1, Mel270, Mel285, MP38, and MP46;
• Metastatic UM cell lines: OMM1, OMM2.3, OMM2.5, MM28, and MM66;
• BAP1-positive UM cell lines: 92.1, Mel270, Mel285, OMM1, OMM2.3, OMM2.5, and MM66;
• BAP1-negative UM cell lines: MP38, MP46, and MM28.

### Binding and Uptake of AU-011

Cells were seeded in 96-well plates (Greiner) at 5000 cells per well and allowed to attach for 24 hours. They were then incubated with different concentrations (range from 3 pM to 900 pM) of AU-011 during indicated time periods at 4°C (binding) or 37°C (uptake). After the indicated time period, cells were washed with phosphate-buffered saline (PBS) and fixed with 4% formalin (J.T. Baker, Landsmeer, The Netherlands) for 30 minutes. The cells were washed 3 times and reconstituted in 100 uL FACS buffer (PBS with 0.5% bovine serum albumin [BSA] and 0.02% sodium azide). The fluorescence of AU-011 was then measured by LSR-II (BD Biosciences, San Jose, CA, USA) in the APC-Cy7 channel.

### Fluorescence Microscopy

Mel270 cells were seeded in 8-chamber polystyrene-vessel tissue-culture glass slides (Corning, Kennebunk, ME, USA) at 3000 cells per chamber and allowed to attach for 24 hours. Cells were incubated with 300 pM AU-011 for 4 hours at 4°C or 37°C. Slides were washed with PBS and incubated with a cell surface marker (CD44-FITC; Thermofisher) at 4°C for 30 minutes. Then, the slides were washed 3 times with PBS and fixed with 1% formalin (J.T. Baker) for 10 minutes at room temperature. The cells were washed again with PBS and stained with DAPI (4',6-diamidino-2-phenylindole, Sigma, St. Louis, MO, USA) at 5 µg/mL for 5 minutes. Slides were again washed 3 times with PBS, after which coverslips were mounted on the glass slides using Mowiol mounting medium (Sigma) supplemented with 2.5% DABCO and sealed with nail polish. Slides were imaged on a Leica SP8 fluorescence microscope.

### In Vitro Cytotoxicity and EC_50_ Determination

Light activated AU-011 cytotoxicity was measured by flow cytometry. First, 50,000 UM cells were seeded in 24-well plates (Corning) in medium and allowed to attach overnight at 37°C and 5% CO2 in an incubator. Cells were incubated with AU-011 (concentration range 3 pM to 900 pM, with most test conditions at 300 pM). Then, cells were washed three times with PBS and supplied with fresh medium. Immediately after, the cells were irradiated at a total light dose (fluence) of 25 J/cm^2^ and a light intensity (fluence rate) of 600 mW/cm^2^ using a 690 nm LED diode laser (CNI Laser, Changchun, China), unless indicated otherwise. At 24 hours after treatment, the tumor cells were stained with Annexin V-FITC (Thermo Fisher, Eugene, OR, USA) at 2.5 µL per sample and 0.25 mg/mL DAPI (Sigma) in Annexin V-binding buffer (Thermo Fisher), followed by analysis using an LSR-II (BD Biosciences, San Jose, CA, USA). The EC_50_ of the UM cell lines was measured by flow cytometry.

### RNA Expression of Damage Associated Molecular Patterns

RNAseq data were acquired from Institut Curie (Paris, France). A panel of UM cell lines (as described in “cell lines and cell culture”) was used for the RNAseq experiment; UM cells without treatment were used for RNA isolation. The isolation of total RNA was done with a NucleoSpin Kit (Macherey-Nagel, Düren, Germany). Based on the manufacturer's instructions (Invitrogen, Carlsbad, CA, USA), cDNA synthesis was conducted with MuLV Reverse transcriptase and under the quality assessments by an Agilent 2100 Bioanalyzer (Santa Clara, CA, USA). Libraries were then constructed and sequenced. For the data analysis, the fastq were mapped against the human reference genome (hg19) using TopHat (version 2.0.6). Gene expression data were obtained using FeatureCounts from the subread package (version 1.5.0). After filtering out genes with low expression or no variation, DESeq2 (R package) was used to normalize gene expression data.

### Measurement of CRT and HSP90 at the Cell Surfaces

UM were treated by AU-011 PDT or three cycles of freeze-thawing (freeze cells at −20°C for 1 hour and then thawed at 37°C for 1 hour, 3 cycles). After 24 hours of incubation, the cells were collected and washed with fluorescent activated cell sorting (FACS) buffer, and then resuspended in FACS buffer with recombinant PE anti-calreticulin antibody (Abcam, Cambridge, UK), recombinant Alexa 488-HSP90 antibody (Abcam), and DAPI (Sigma-Aldrich, Zwijndrecht, The Netherlands) for 30 minutes at 4°C. Finally, the samples were analyzed by flow cytometry on an LSR-II (BD Biosciences).

### Statistical Analysis

Statistical analyses were performed in GraphPad Prism version 9.0 for Windows (GraphPad Software, La Jolla, CA, USA). Student's unpaired two-tailed *t*-test was performed to compare two experimental groups for the analysis of DAMPs. The RNA expression of DAMPs was compared using the Mann–Whitney *U* test. EC50 was calculated using a nonlinear regression analysis. Unless otherwise stated, data are shown as the mean ± SEM of the data from three independent experiments. Statistical differences were considered significant at *P < 0.05, **P < 0.01, and ***P < 0.001.

## Results

### Cellular Binding, Uptake, and Location of AU-011

**Figure 1.**
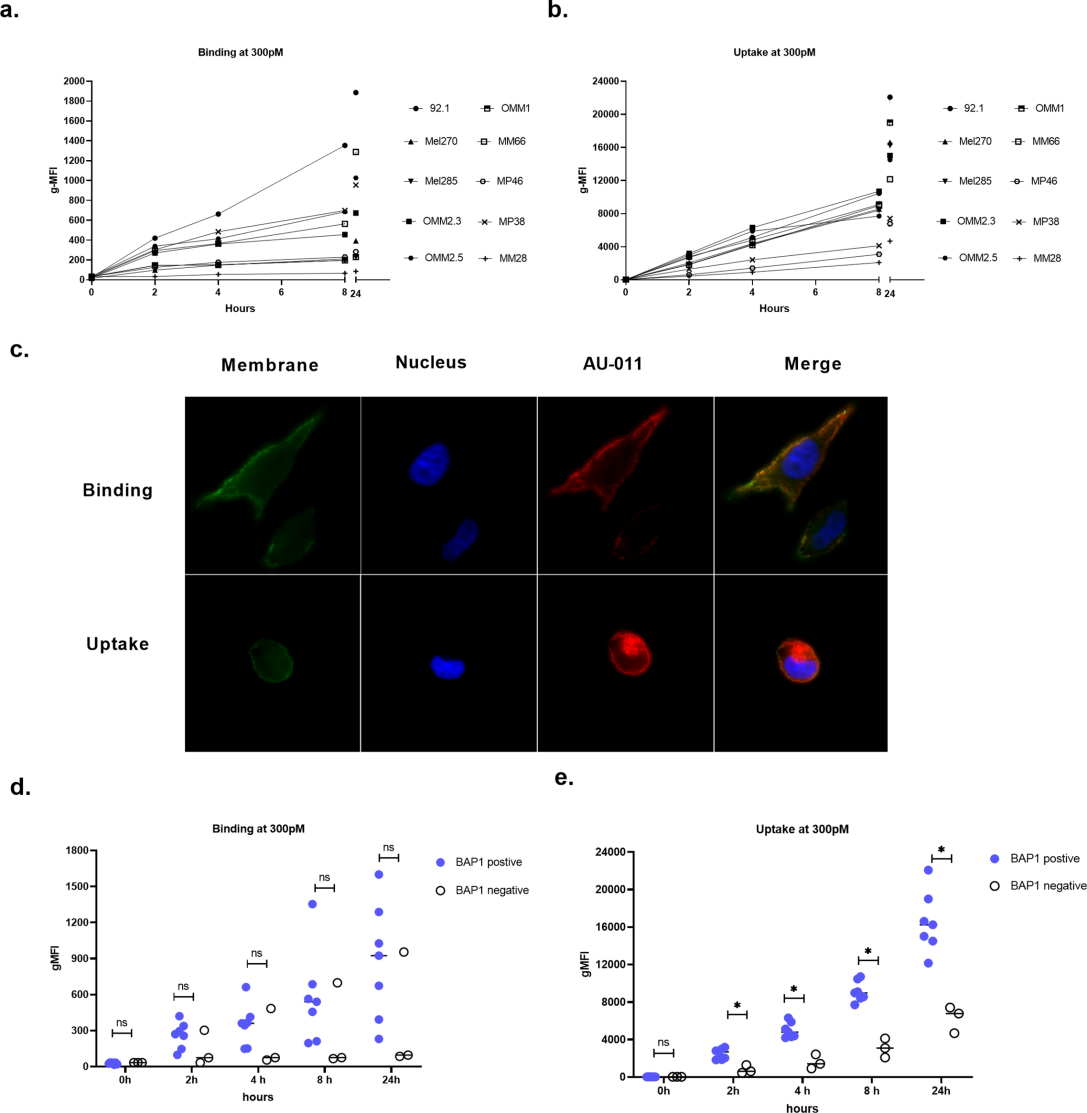
**AU-011 binding, uptake and subcellular location in vitro.** (**a**) Binding and (**b**) uptake of AU-011 at indicated concentrations in 10 UM cell lines. The graphs show the mean gMFI ± SEM of cells from three independent experiments. (**c**) Location of AU-011 in Mel 270 after staining with CD44-FITC and DAPI imaged by fluorescence microscope. (**d, e**) The binding and uptake of AU-011, comparing BAP1-negative and positive UM cell lines. Student's unpaired two tailed *t*-test was used for statistical analysis, *P < 0.05, **P < 0.01, and ***P < 0.001. Data are presented as the mean ± SD.

First, we confirmed prior work by Kines et al.^9^ that AU-011 can target UM cell lines by assessing its binding and uptake in UM cell lines. These were measured using its geometric mean fluorescent intensity (gMFI). We extended the study to include 10 UM cell lines, 3 of which were BAP1 negative. The intensity of the signal at 37°C (uptake) was significantly higher than at 4°C (binding), showing that at 37°C, AU-011 is not only bound to the cell surface but also localizes inside the cell (Figs. 1a, 1b), (Supplementary Figs. S1 and S2). Indeed, fluorescence microscopy shows that after incubation at 4°C, most AU-011 is located at the cell surface, whereas after incubation at 37°C most AU-011 is in the cytoplasm (Fig. 1c). In all cell lines, binding of AU-011 to the cellular surface on UM cells increased over time, up to at least 24 hours after incubation. A difference was observed between BAP1-positive and BAP1-negative UM cell lines, whereby BAP1-negative UM cell lines displayed a markedly reduced uptake compared to BAP1-positive UM cell lines (Fig. 1e). As incubation for 4 hours at 300 pM resulted in substantial accumulation of AU-011, this condition was used for the following in vitro experiments, unless indicated otherwise.

### Light-Activated AU-011 Induces Cell Death in UM Cell Lines

**Figure 2.**
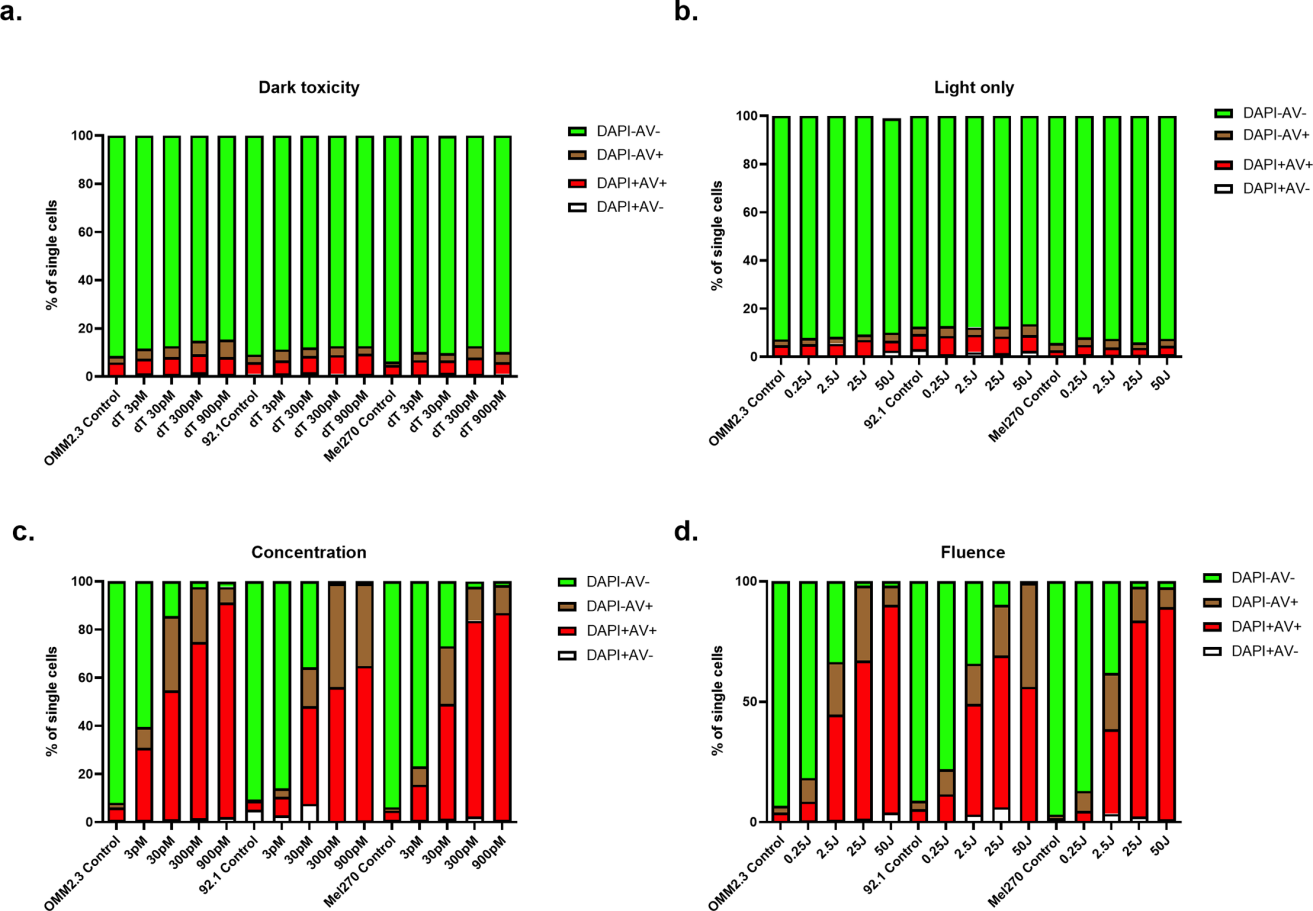
**Light-activated AU-011 induced cell death in vitro**. (**a**) Dark toxicity and (**b**) light toxicity of AU-011 in OMM2.3, 92.1, and Mel270 cells. The effect of (**c**) concentration and (**d**) fluence on in vitro cytotoxicity of AU-011 on OMM2.3, 92.1, and Mel270 cells, measured by flow cytometry after staining with Annexin V and DAPI. DAPI indicates late apoptosis cells while AV (Annexin V) staining indicates the early apoptosis cells.

To determine the sensitivity of UM cell lines to AU-011 after near infrared (NIR) light activation, we evaluated the toxicity of AU-011 in the absence of light (dark toxicity) and light only by incubating cells with AU-011 only or irradiation with light only, respectively. As expected, the dark toxicity of AU-011 at concentrations of 3 to 900 pM (Fig. 2a) and the toxicity induced by NIR light irradiation alone at a fluence range of 0.25 to 50 J/cm^2^ (Fig. 2b) were found to be minimal.

Subsequently, UM cells were incubated with AU-011 and then irradiated with 690 nm light. Three variables were taken into account to determine the antitumor efficiency: fluence, concentration, and fluence rate. The cytotoxicity of AU-011 was enhanced with increasing concentrations of AU-011 (Fig. 2c) and with increasing fluence (Fig. 2d). However, this was not observed when evaluating different fluence rates, for which all conditions displayed strong cytotoxicity (Supplementary Fig. S3). These data show that light-activated AU-011 induced near-complete UM cell death in vitro.

### EC50 Values of a Panel of Uveal Melanoma Cell Lines With Light-Activated AU-011

Next, we determined the EC_50_ values of AU-011 for the different UM cell lines by flow cytometry. UM cell lines with EC_50_ values that display picomolar ranges (15–40 pmol/L; see the Table). These data show that light activated AU-011 induces strong cytotoxicity in most UM cells in vitro. The higher EC_50_ in BAP1-negative cell lines (*P* < 0.01) is consistent with the results of their lower uptake of AU-011.

**Table.** The Potency EC_50_ Values (PMOL/L) of AU-011 on UM Cell Lines as Determined By Flow Cytometry

	Killing	Derived From	BAP1 Protein Expression
OMM2.3	14.9 ± 3.0	M	**+**
Mel270	17.3 ± 3.7	P	**+**
Mel285	18.7± 4.0	P	**+**
92.1	19.7 ± 3.8	P	**+**
OMM1	24.4 ± 3.3	M	**+**
MM66	25.2 ± 2.7	M	**+**
OMM2.5	29.7 ± 3.6	M	**+**
MP38	33.1 ± 2.0	P	**–**
MP46	36.4 ± 3.5	P	**–**
MM28	40.2 ± 3.3	M	**–**

P, primary; M, metastasis.

Values represent mean pmol/L ± SEM, obtained from experiment performed in triplicate.

### Membrane Exposure of Damage-Associated Molecular Patterns After Treatment

In addition to inducing direct cytotoxicity, light-activated AU-011 may trigger an immune response by causing immunogenic cell death. This phenomenon is accompanied by increased cell surface exposure of DAMPs, such as CRT and HSP90. These DAMPs can initiate an acute inflammation in the tumor area that may lead to the induction of an antitumor immune response.

**Figure 3.**
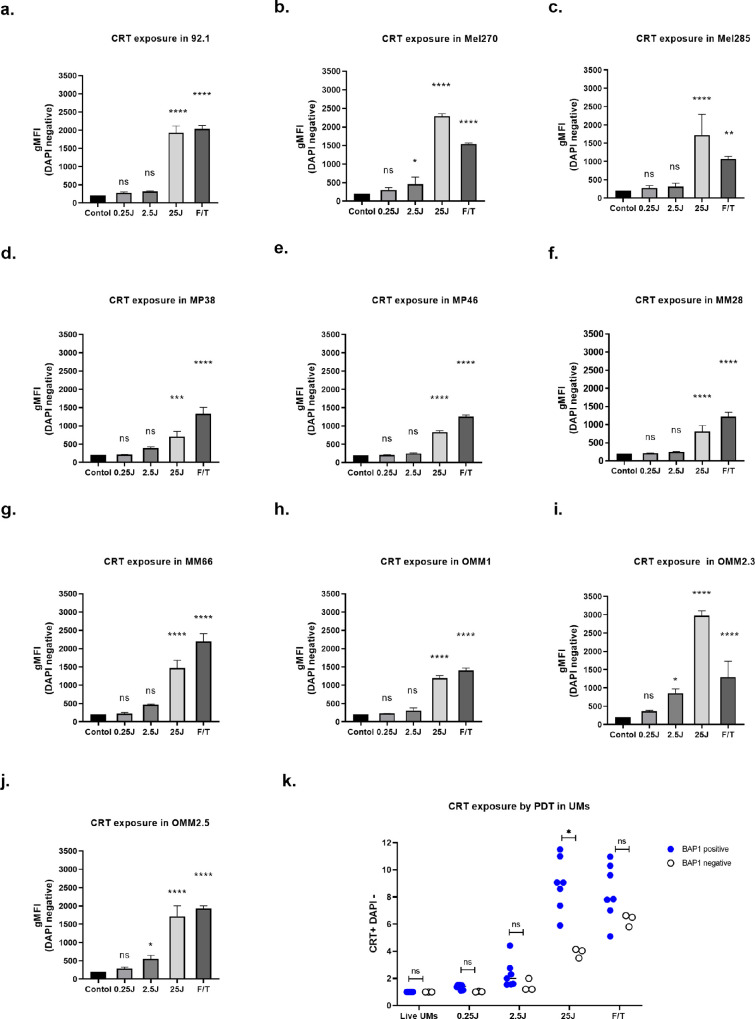
**Cell surface exposure of CRT following AU-011 treatment.** (**a-j**) Level of exposure of CRT on the cell surface as determined by flow cytometry on 10 uveal melanoma cell lines on DAPI-negative cells. UM cells were treated with AU-011 followed by light activation with 0.25, 2.5, and 25 J/cm^2^ or were left untreated and analyzed 24 hours later. As a positive control, cells were frozen (−20°C) and thawed (37°C) three times. Data represented as the relative mean fluorescence intensity. (**k**) Comparison of CRT exposure induced by 25 J/cm^2^ of light activation between BAP1-positive and BAP1-negative (MM28, MP38, and MP46) UM cell lines. One-way ANOVA followed by Tukey's posthoc test was used for statistical analysis; *P < 0.05, **P < 0.01, and ***P < 0.001. Data are presented as the mean ± SD.

**Figure 4.**
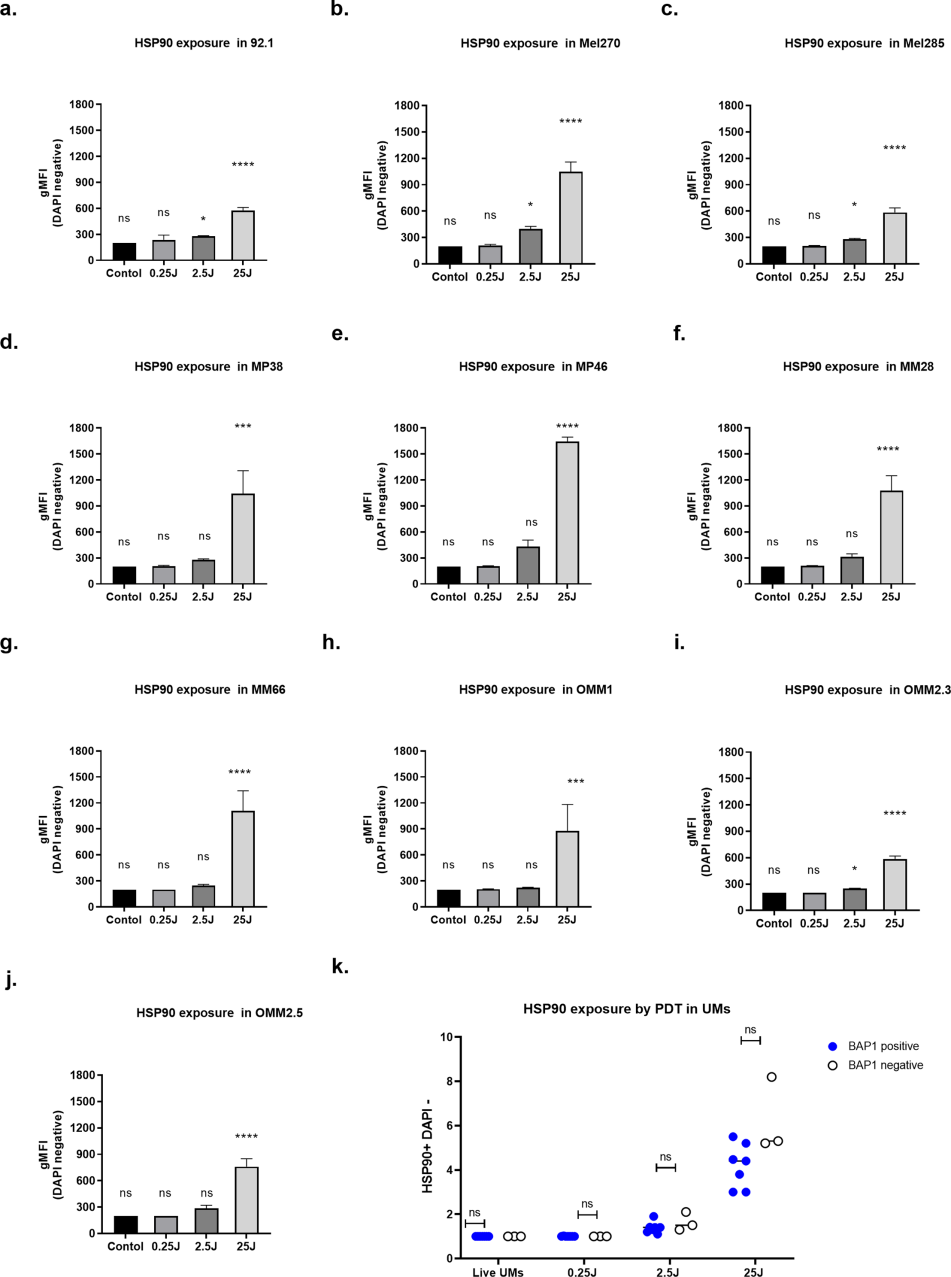
**Induced cell surface exposure of HSP90 following AU-011 treatment**. (**a-j**) Level of exposure of HSP90 on the cell surface as determined by flow cytometry on ten uveal melanoma cell lines. UM cells were treated with AU-011 followed by light activation with 0.25, 2.5, and 25 J/cm^2^ or were left untreated and analyzed 24 hours later. (**k**) Comparison of HSP90 exposure induced by 25 J/cm^2^ of light activation between BAP1-negative and -positive UM cell lines. The graphs show the mean gMFI ± SEM of cells from three independent experiments. One-way ANOVA followed by Tukey's post hoc test was used for statistical analysis; *P < 0.05, **P < 0.01, and ***P < 0.001. Data are presented as the mean ± SD.

Evaluation of CRT and HSP90 exposure induced by light-activated AU-011 in UM cell lines was performed using flow cytometry. CRT exposure was significantly enhanced 24 hours after light activation, with a further increase at higher fluence. It is noteworthy that the overexposure of CRT at the cell surface of UM cells after light activation was enhanced compared to freeze-thawed cells, which were used as a positive control (Figs. 3a-j). After treatment with 25 J/cm^2^, the induction of CRT was significantly lower in BAP1-negative cell lines than in BAP1-positive cell lines (Fig. 3k). When looking at HSP90 exposure after treatment, we saw an increase in HSP90 exposure on all 10 cell lines 24 hours after light activation of AU-011 at 25 J/cm^2^ (Fig. 4). For HSP90, there was no significant difference between BAP1-positive and BAP1-negative cell lines.

**Figure 5.**
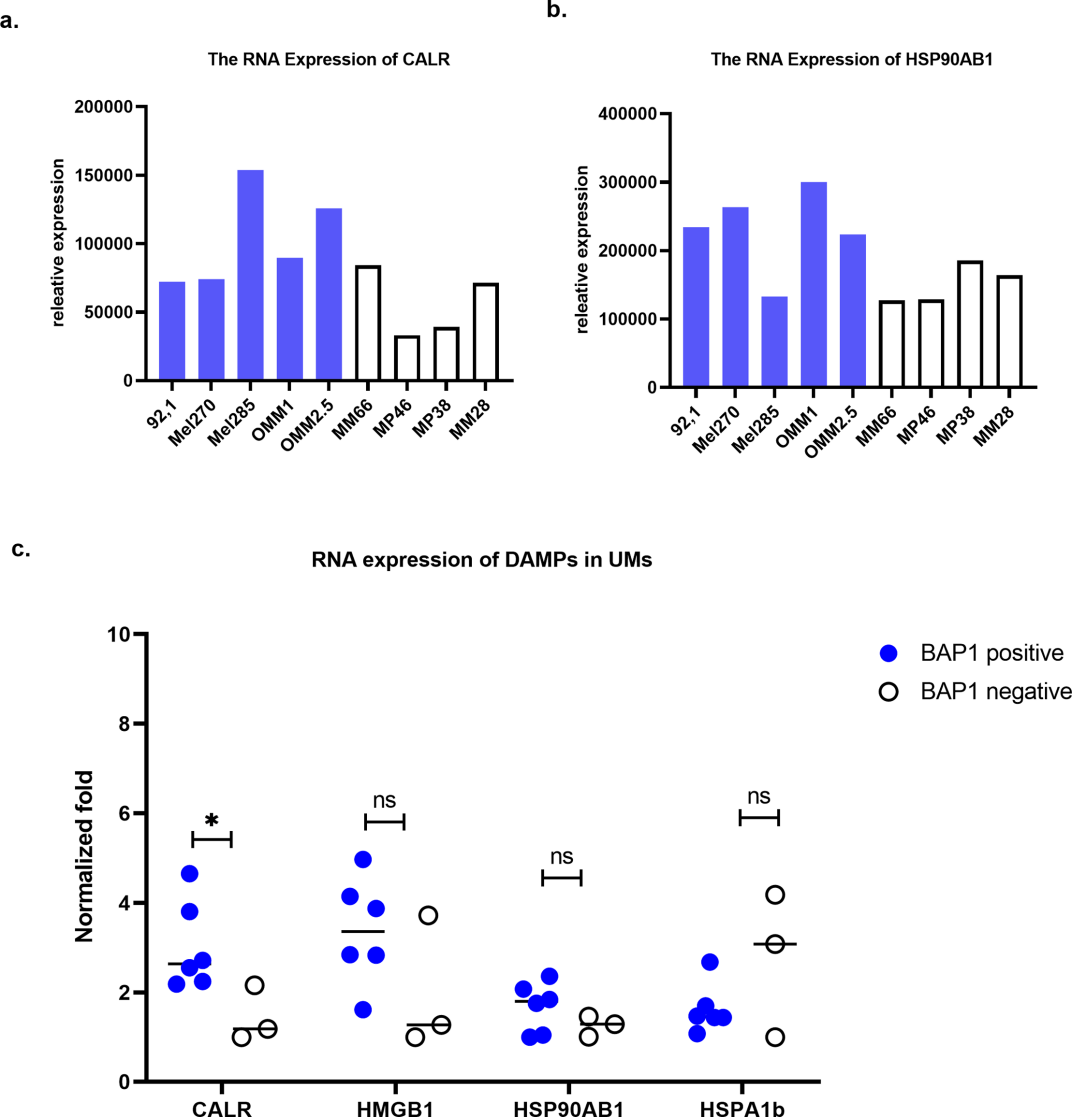
**RNA expression of DAMPs in** **10** **UM cell lines determined by RNAseq**. (**a**) CRT expression (CALR gene), (**b**) HSP90 expression (HSP90AB1 gene), (**c**) comparison of RNA expression of DAMPs between BAP1- positive and BAP1-negative UM cell lines. HSPA1b is the gene for HSP70. Using the Mann-Whitney *U* test, statistical differences were significant at *P < 0.05, **P < 0.01, and ***P < 0.001. Data are presented as the mean ± SD.

As we noticed differences between cell lines, we analyzed the basic RNA expression of DAMPs in cultured UM cell lines as determined by RNAseq (Figs. 5a, 5b). The data showed that baseline CRT expression (CALR gene) varied between cell lines and was higher in BAP1-positive cell lines than in BAP1-negative cell lines (*P* < 0.05). There was no significant difference in HMGB1, HSP90 (HSP901A gene, very low expression), or HSP70 (HSPA1b gene) expression between BAP1-positive and negative cell lines (Fig. 5c).

Taken together, the data show that NIR light activation of AU-011 induces CRT, as well as HSP90 membrane exposure, which may be an indication of its ability to induce immunogenic cell death in UM cells. CRT expression is higher in BAP1-positive cell lines than BAP1-negative cell lines, whereas, in addition, AU-011 treatment stimulates a stronger CRT exposure in BAP1-positive UM cell lines than BAP1-negative UM cell lines (see Fig. 3k).

## Discussion

We investigated the tumor cell killing and potential immunostimulatory capacity of AU-011 treatment using a panel of ten human UM cell lines to provide supportive evidence for combining this treatment with immunotherapy in future in vivo experiments and clinical trials. We demonstrated that AU-011 treatment can induce strong cytotoxicity in UM cell lines. Furthermore, we found that NIR light-activated AU-011 could enhance the membrane exposure of DAMPs on UM cell lines indicating that AU-011 treatment is capable of inducing immunogenic cell death. These results suggest that a virus-like drug conjugate like AU-011 and immunotherapy treatment, such as ICI, may function as a potential combination treatment in malignant tumors, such as UM.

The binding and uptake experiments show that AU-011 can bind to and is taken up by UM cells, leading to effective cell death after irradiation. Our data show that the BAP1-negative UM cell lines display a lower uptake of AU-011 and a higher EC_50_ than the BAP1-positive cell lines. BAP1 (BRCA1-associated protein-1) is a ubiquitin carboxy-terminal hydrolase that has been shown to be a multifunctional tumor suppressor gene and is involved in cellular metabolism and DNA repair by regulating gluconeogenesis via PGC1 stabilization, suppressing mitochondrial respiration.^36^^–^^38^ As the process of uptake of AU-011 consumes ATP, this may explain the reduced uptake of AU-011 in BAP1-negative UM cells.^39^^,^^40^

Regardless of BAP1 mutation or other mutations in UM cell lines, the observation that the EC_50_ of AU-011 treatment is in the picomolar range indicates that most UM cells are sensitive to AU-011 treatment, as previously shown by Kines et al.^9^ This notion may be attributed to the lack of resistance mechanisms against the targeted necrosis-induced cell death,the exposure of tumor neoantigens and the unique characteristics of AU-011 that make it a treatment that is potentially genetic mutation agnostic. As we analyzed a larger set of cell lines, we were struck by the finding that BAP1-negative cell lines had a higher EC_50_ than BAP-1 positive cell lines.

Our data show that AU-011 treatment not only causes UM cell death but also induces markers of immunogenic cell death, as characterized by the cell surface exposure of DAMPs, such as CRT and HSP90.^41^^,^^42^ This indicates the potential immunostimulatory capacity of light-activated AU-011 using human UM tumor cell lines. The exposure or release of DAMPs can recruit neutrophils, macrophages, and dendritic cells into the tumor, triggering a local inflammatory response.^43^^,^^44^ CRT and HSP90 can serve as activation signals for phagocytic cells, enhancing phagocytosis of tumor-associated antigens. Here, we noticed a difference in relation to BAP1 as well: untreated BAP1-negative UM cell lines have a lower CRT expression compared to BAP1-positive UM cell lines. Moreover, CRT exposure induced by light-activated AU-011 was lower in BAP1-negative UM cell lines than in BAP1-positive UM cell lines, potentially providing them with a lower immunostimulatory ability. It was recently reported that BAP1 is involved in the cellular calcium release, which functions as a tumor suppressor in the cytoplasm.^45^ However, the RNA expression in untreated UMs and exposure of HSP90 induced by light-activated AU-011 shows no difference between BAP1-positive and negative groups, indicating that AU-011 treatment may induce a higher immunogenicity in all UM through other DAMPs.

Our data provide a potential strategy to induce effective cytotoxicity and enhance the immunostimulatory capacity for UM in vitro and possibly in vivo. The aim for first line targeted therapies for UM is to conserve the eye and preserve vision, as well as prevent outgrowth of metastases. AU-011 can specifically target UM cells and has minimal off-target toxicity.^9^ Immunogenic cell death can serve as an adjuvant for immunotherapy to establish a systemic immune response. Loss of BAP1 expression is associated with lower expression and AU-011-induced membrane exposure of CRT, which may influence responsiveness to immunotherapy through CRT. In the context of the eye, light-activated AU-011 may locally overcome the immune privilege which tends to suppress local immune responses. Our data with multiple UM cell lines and inducing DAMPs support future studies that investigate the combination of AU-011 treatment with immunotherapy in vivo and may inspire the initiation of clinical trials to treat the primary tumor and associated metastatic disease.

## Supplementary Material

Supplement 1
