# Supplementary material for: In Vitro Testing of the Virus-Like Drug Conjugate Belzupacap Sarotalocan (AU-011) on Uveal Melanoma Suggests BAP1-Related Immunostimulatory Capacity
Source: Invest Ophthalmol Vis Sci. 2023 Jun 5;64(7):10. doi: 10.1167/iovs.64.7.10 (PMC10246758; doi:10.1167/iovs.64.7.10)
Supplement: Supplement 1 [file iovs-64-7-10_s001.pdf]

Supplementary files

In vitro testing of virus like drug conjugate belzupacap sarotalocan(AU-011) on uveal melanoma suggests BAP1-related immunostimulatory capacity

Sen Ma[1], Ruben Huis In't Veld[2,3], Alexander Houy[4], Marc-Henri Stern[4] , Cadmus Rich[4], Ferry Ossendorp[2], Martine Jager[1]*

[1] Department of Ophthalmology, Leiden University Medical Center (LUMC), Albinusdreef 2, 2333 ZA Leiden, the Netherlands

[2] Department of Radiology, Leiden University Medical Center (LUMC), Albinusdreef 2, 2333 ZA Leiden, the Netherlands

[3] Department of Immunology, Leiden University Medical Center (LUMC), Albinusdreef 2, 2333 ZA Leiden, the Netherlands

[4] Inserm U380, DNA Repair and Uveal Melanonoma (D.R.U.M.), Equipe labelliseé par la Ligue Nationale Contre le Cancer, Institut Curie, PSL Research University, Paris, France

[5] Aura Biosciences, Inc., Cambridge, MA, US

* Correspondence: M.J. Jager, Department of Ophthalmology, Leiden University Medical Center (LUMC), Albinusdreef 2, 2333 ZA Leiden, the Netherlands; e-mail M.J.Jager@lumc.nl

Supplementary materials.

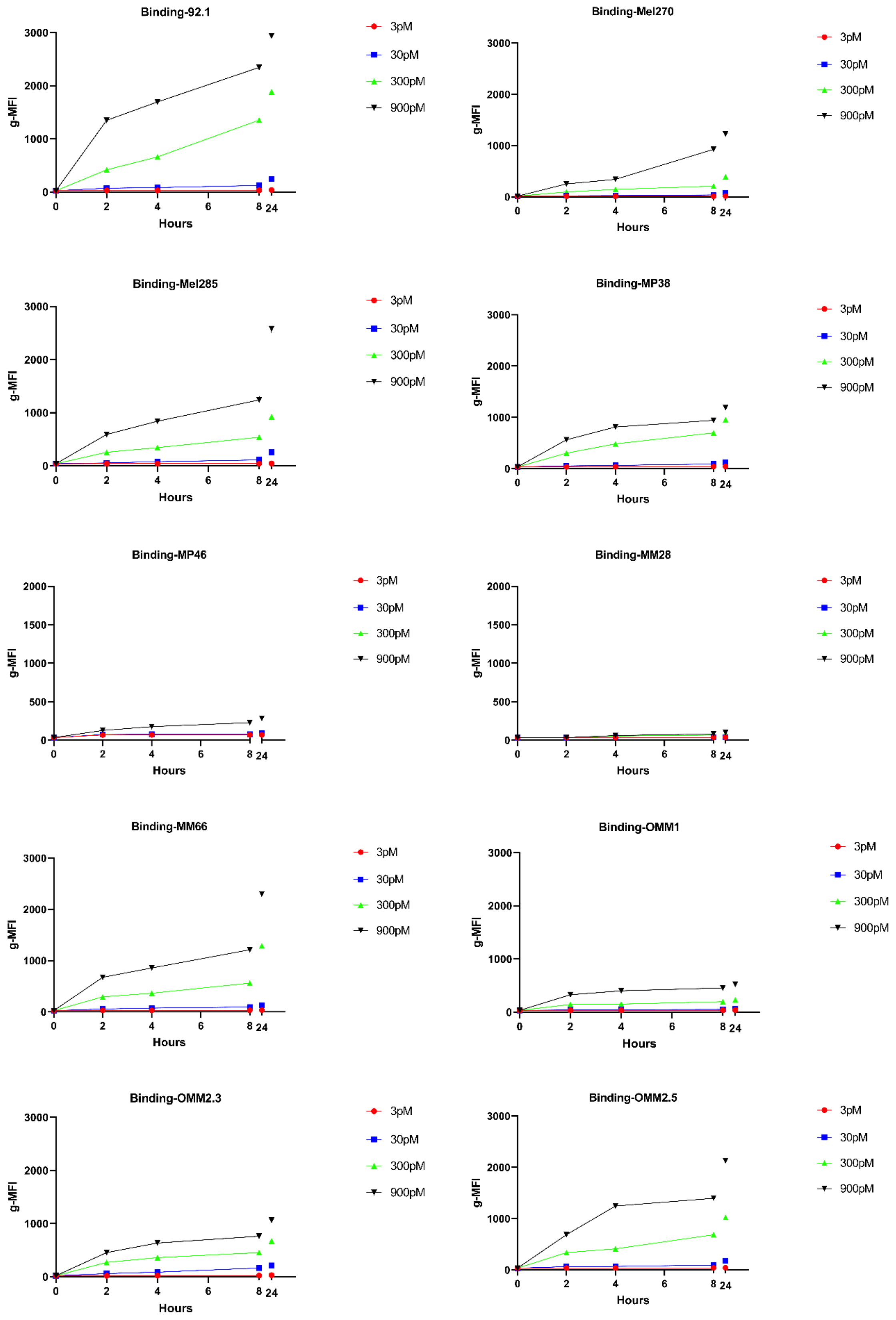

Fig S1. AU-011 binding at indicated concentration in a panel of uveal melanoma cell lines over time in 4°C by flow cytometry in APC-Cy7 channel.

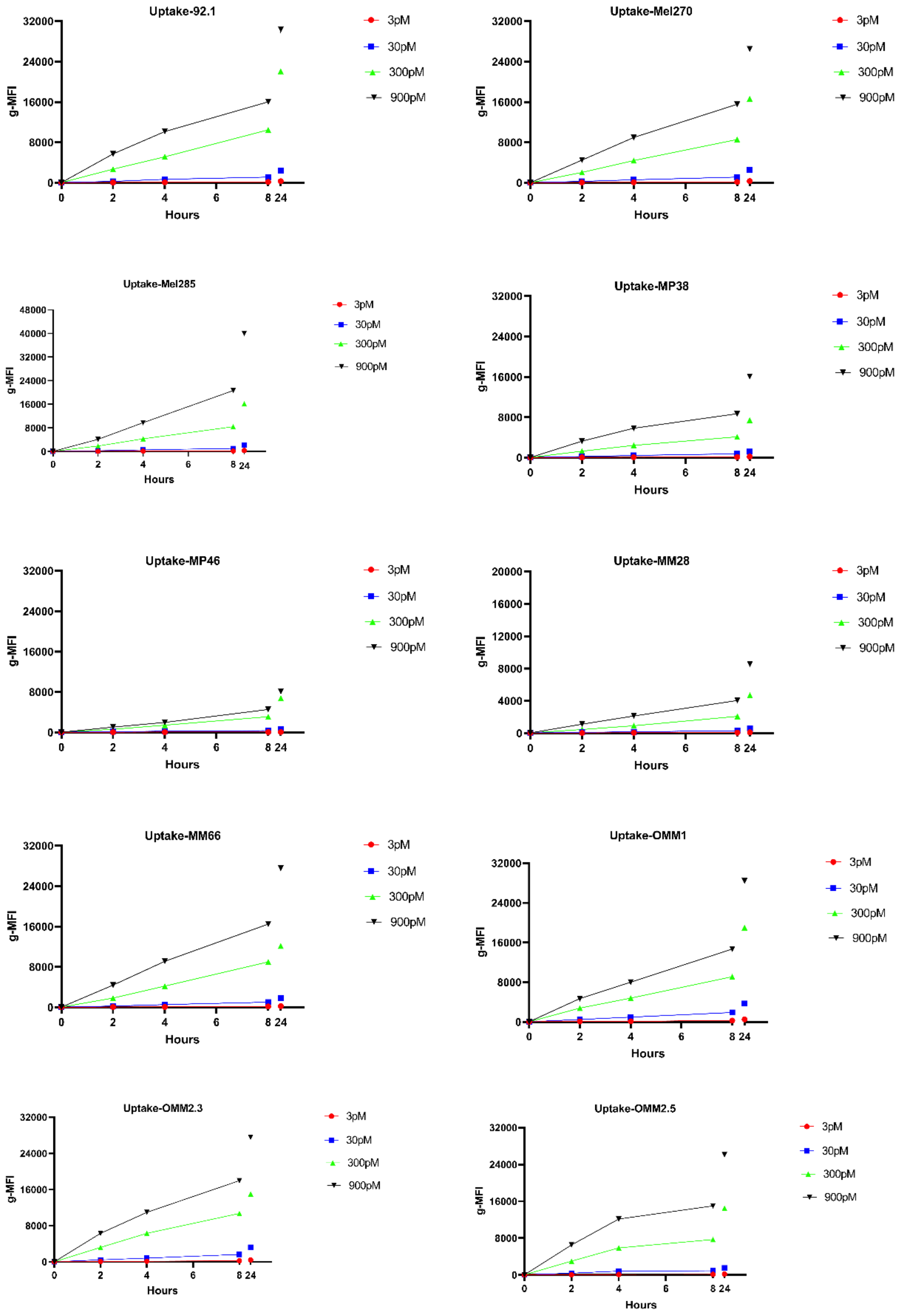

Fig S2. AU-011 uptake at indicated concentration in a panel of uveal melanoma cell lines over time in 37°C by flow cytometry in APC-cy7 channel.

a.

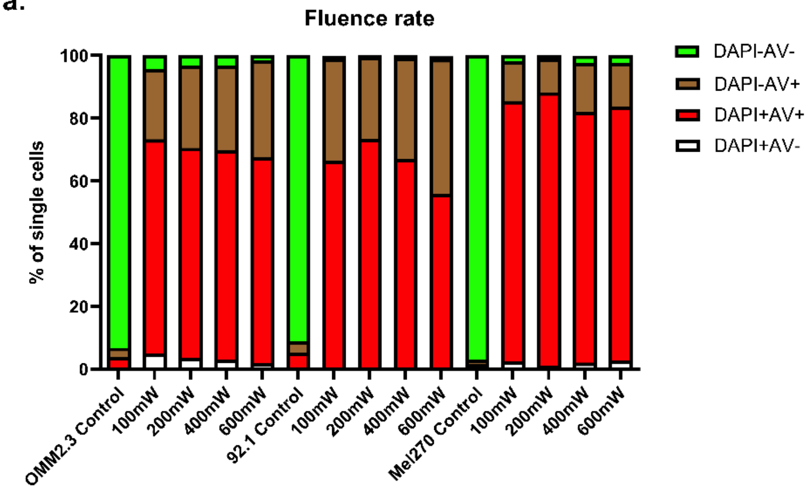

b.

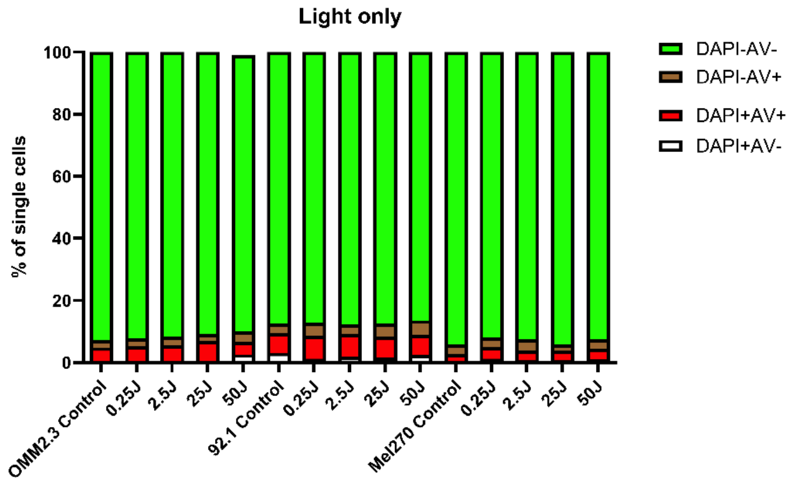

Fig S3. Light activated AU-011 and light only (without AU-011 incubation) induced cell death in vitro.

The effect of fluence rate a) and light irritation only b) in vitro cytotoxicity of AU-011 treatment on uveal melanoma cells, determined by flow cytometry after staining with Annexin V and DAPI. DPAI indicates late apoptosis cells while AV(Annexin V) staining indicates the early apoptosis cells.
